# Effect of Crisis Plans on Admissions and Emergency Visits: A Randomized Controlled Trial

**DOI:** 10.1371/journal.pone.0091882

**Published:** 2014-03-19

**Authors:** Asia Ruchlewska, Andre I. Wierdsma, Astrid M. Kamperman, Mark van der Gaag, Renee Smulders, Bert-Jan Roosenschoon, Cornelis L. Mulder

**Affiliations:** 1 Epidemiological and Social Psychiatric Research institute, Erasmus MC, Rotterdam, The Netherlands; 2 BavoEuropoort, Centre for Mental Health Care, Rotterdam, The Netherlands; 3 Parnassia Psychiatric Institute, The Hague, The Netherlands; 4 VU University and EMGO Institute for Health and Care Research, Amsterdam, The Netherlands; 5 Landelijk Crisiskaart (O)GGZ Informatie en ondersteuningspunt, Utrecht, The Netherlands; The University of Queensland, Australia

## Abstract

**Objective:**

To establish whether patients with a crisis plan had fewer voluntary or involuntary admissions, or fewer outpatient emergency visits, than patients without such a plan.

**Design:**

Multicenter randomized controlled trial with two intervention conditions and one control condition.

**Participants:**

Adult outpatients diagnosed with psychotic or bipolar disorder who had experienced at least one psychiatric crisis in the previous two years.

**Intervention:**

Two types of advance statement were used: (1) a crisis plan formulated by the patient with the help of a patient advocate (Patient Advocate Crisis Plan: PACP); and (2) a crisis plan developed together with the clinician (Clinician-facilitated Crisis Plan: CCP).

**Outcome:**

The percentages of patients admitted voluntarily or involuntarily (on an emergency basis or by court order), and the percentage who made outpatient emergency visits over an 18-month follow-up period.

**Results:**

A total of 212 patients were included: 69 in the PACP condition, 70 in the CCP condition, and 73 in the control condition. No effects of the two interventions were found on the numbers of voluntary admissions, involuntary admissions and emergency visits. Regarding involuntary admissions, there was no significant effect on emergency admissions, which were 17% (12/69) in the PACP condition, 10% (7/70) in the CCP condition, and 19% (14/73) in the control condition. There was a significant effect on planned court-ordered admissions, with 16% (11/69) in the PACP condition, 10% (7/70) in the CCP condition, and 26% (19/73) in the control condition. Finally, the interventions had no effect on outpatient emergency visits, with 32% (22/69) in the PACP group, 31% (22/70) in the CCP group, and 34% (25/73) in the control group.

**Conclusions:**

Crisis plans may be an effective intervention for reducing court-ordered admissions in patients with psychotic and bipolar disorders.

**Trial registration:**

Current Controlled Trails NTR1166.

## Introduction

Voluntary and involuntary admissions have a strong impact on patients and their relatives [1], [2]. In some countries, including the Netherlands, the numbers of admissions have increased over recent years [3].

Psychiatric advance statements may prevent involuntary admissions. However, only few studies investigated the effects of advance statements: Henderson et al. [4] showed that involuntary admissions may be prevented by joint crisis plans, a form of psychiatric advance statement. However, a multicentre study using the same type of advance statement could not replicate this result [5]. Another study [6] used a different form of advance statement and also showed no effects on the number and type of admissions.

Advance statements aim to increase patients' self-determination at times when they are incapable of specifying their treatment preferences, which sometimes happens during involuntary admission. These statements have also been reported to help prevent psychiatric crises [7]. While it is not known which factors influence their effects, we previously hypothesized that the effects may be mediated by the service engagement, social support, insight and the quality of the working alliance [8].

Different types of advance statement coexist, each characterised by the way they are created. For example, a mental-healthcare provider may be involved in making a statement, or it may be facilitated independently [9].

In the Netherlands there are two types of advance statement: a crisis plan that is created together with a patient advocate (Patient Advocate Crisis Plan: PACP), and one that is made with the clinician (Clinician-facilitated Crisis Plan: CCP). Each type contains the description of crisis prevention and practical information for handling future psychiatric emergencies. The information is summarized on a small card, the ‘crisis card’, which users carry with them at all times. Crisis plans are developed on a voluntary basis. As they are not legally binding, actual treatment – during involuntary admission, for instance – may diverge from the preferences or refusals stated in the plan.

The primary aim of the present study was to examine whether a crisis plan facilitated by the patient advocate or the clinician could reduce voluntary admissions, involuntary admissions, and emergency visits. We also investigated the possible associations between the effects of the crisis plans in relation to service engagement, social support, insight and the quality of the therapeutic alliance.

## Methods

The protocol for this trial and supporting CONSORT checklist are available as supporting information; see Checklist S1, Protocol S1 and Protocol S2. Research data is available for secondary analysis and may contribute to larger datasets of routinely collected outcome data or service user data. Data will be shared in anonymized form. Data archiving and curating is executed within the ethical, legal and institutional regulatory framework of the Erasmus Medical Center Rotterdam.

### Ethical approval

The study protocol, information brochure and informed consent form were approved by the Dutch Union of Medical-Ethic Trail Committees for mental health organizations (registration number 7.109, CCMO-nr NL 16818.097.07).

### Participants and setting

Participants in the study were outpatients aged between 18 and 65 years who had a diagnosis of schizophrenia or other psychotic disorder, and bipolar disorder II, and who had had at least one emergency outpatient contact with the mental health services, or one voluntary or involuntary admission over the previous two years. They were recruited from 12 *Assertive Community Teams* and *Illness Management & Recovery* teams in Rotterdam, the Netherlands. There were four exclusion criteria: having a somatic illness that caused a psychotic disorder, the inability to give informed consent because of mental incapacity, an insufficient command of the Dutch language, and already having a crisis plan or another type of advance statement.

### Recruitment of participants and data collection

Originally the planned start date for patient recruitment was October 15, 2007. Due to logistical delays patient recruitment began in January 2008 and ended in March 2011. Candidate participants were selected from the clinicians' caseloads by the clinician and the researcher on the basis of the inclusion and exclusion criteria. The patients selected received an information letter about the study from their clinicians, who requested the patients' permission to be contacted by an independent researcher. The interviewer explained the research goals and randomisation procedure. The baseline interview followed the provision of written informed consent. The second interview with the patient was scheduled eighteen months after the baseline measurement.

### Interventions

#### Patient Advocate Crisis Plan: PACP

Patient advocacy is a lay specialization in health care. Patient advocates are often (former) psychiatric patients, trained to represent the interests of current patients in mental health care. This is done by providing patients with information, advice and support regarding mental health and health care, and their legal position and rights as a patient. Patient advocates can also help with filing complaints and mediate between patient and service provider with finding solutions. The two participating patient advocates in this study were social workers with over fifteen years of work experience in the mental health services; one was also an expert by experience. Both worked for a patient organization. Their main focus was the creation of crisis plans together with the patients.

After the randomization, the patient advocate made an appointment. During the first meeting, the advocate discussed the procedure with the patient and collected information for the crisis plan. Crises-precipitating factors were discussed and strategies for preventing crises were developed. After this meeting, the advocate prepared the first concept of the plan. Then, the patient, supported by the advocate, negotiated with his or her clinician about what to do when the first signs of a crisis develop and what his or her wishes are about what to do in times of crisis. After completion of the plan, it was signed by the patient's psychiatrist, the clinician (mostly psychiatric nurses) and other people (e.g. the partner, friends or family) involved in the crisis plan. The final step was to summarize the plan on a crisis card, which was then handed to the patient.

#### Clinician facilitated Crisis Plan: CCP

In the CCP condition, after randomization the researcher explained the structure of the intervention to the clinicians. The clinicians (mostly psychiatric nurses) composed the crisis plan as part of the patients' regular treatment. As in the PACP condition, crises-precipitating factors were discussed and strategies were developed for preventing them. The patient and his or her clinician formulated the content of the crisis plan together. The procedure contained several stages: the preparation and formulation of the crisis plan, an informed discussion, and the collection of signatures of everyone involved in the development process (e.g. the partner, friends or family). The final step was to summarize the plan on a crisis card, which was then handed to the patient.

The content of the crisis plan has to be evaluated annually or more frequently if necessary. All crisis plans were included in the patients' records and in the electronic records of all emergency psychiatric services with which the patient might come into contact during a crisis.

### Structured monitoring

During the study we registered the respective amounts of time needed to complete the PACP and the CCP. In each condition, the researcher (AR) monitored the process whereby the crisis plans were drawn up. To remind the clinicians to finish the plan, the researcher needed to undertake a mean of five actions (i.e. e-mails or telephone calls; SD = 3) in the CCP condition. In the PACP condition, no reminders were necessary in order to finish the plan. Similar problems with the implementation of advance statements by clinicians were encountered by Thornicroft et al. [5].

### Primary outcome measures

Primary outcome measures were collected at baseline and over an 18-month follow-up period; they included any voluntary or involuntary admissions to a psychiatric hospital, and any outpatient emergency visit.

The Dutch Act on Special Admissions to Psychiatric Hospitals distinguishes between two types of involuntary admission. The first type involves an emergency involuntary admissions, whereby the city's mayor, advised by an independent physician, decides if hospital admission is required to counter the emergency situation. An acute dangerous situation may involve danger-to-self, usually a suicidal thoughts or behavior, or it may concern aggressive behavior to others or serious public nuisance. Within a five working days, a judge must decide whether the admission is to be continued. The second type of involuntary admission is the common procedure, whereby a judge determines whether legal conditions have been met based on a medical report by an independent psychiatrist. In this case, the dangerousness criteria mostly include self neglect or social breakdown. Both emergency involuntary admissions and court-ordered involuntary admissions are included in our primary outcome measures.

Data were collected from patients' files, checked against the Rotterdam region Psychiatric Case Register [10].

### Patient characteristics

Demographic variables, the histories of previous admissions and emergency visits, and clinical diagnoses were all collected from patients' files. The Health of the Nation Outcome Scales (HoNOS) was used to check for differences in psychosocial functioning [11], [12].

Patient characteristics were assessed through interviews with patients and clinicians. Patients' engagement with the services was measured through the Services Engagement Scale from clinician's perspective [13]. Social support was measured with the Adult Social Report scale [14], and insight was measured with a self-report Insight into Psychosis scale [15]. The therapeutic alliance between the patient and the clinician was measured through the Working Alliance Inventory [16], [17]. See Ruchlewska et al. [8] for a more detailed description of these measures.

### Sample size and power

The sample size required was calculated on the basis of previous studies of the primary outcome variables: voluntary and involuntary admissions [4]. In a pilot study of the effects of crisis cards, the difference between the baseline percentages admitted in hospital and during the year after the intervention was 25% [18]. This difference was 14% in the Henderson's RCT study [4]. On the basis of these two studies we expected a medium effect size. Based on a local study concerning patients seen in emergency psychiatric services, the percentage of patients who were expected to be admitted to psychiatric hospital in the follow-up period was estimated at 30% to 44% [19]. For percentages in this range, a medium effect size (h = .6) corresponds to differences in percentages of about 20% to 25% [20]. At a significance level of p<0.05 (one sided) and power of 90%, we calculated a required sample size of 50 subjects per group. To compensate for respondents lost to follow-up, we decided to increase this to 80 (total 240).

### Randomisation

Randomisation was stratified by treatment team. To ensure the even distribution of the patient groups within each team, we used envelopes containing 12 lots per team. After written informed consent had been obtained, the principal investigator allocated participants randomly into one of the three conditions (PACP, CCP and control condition).

### Statistical analyses

We used Chi-2 tests to assess differences between intervention conditions regarding the number of patients admitted, voluntarily or under the Mental Health Act, and regarding the number of patients in contact with outpatient emergency services. Multiple logistic regression analyses were performed checking for interaction effects and collinearties for all main factors. Model fit was checked using McFadden R2 and diagnostic scatter plots using standardised residuals. Differences between the intervention and control conditions with regard to continuous variables were assessed using Repeated Measure Analyses of Variance or Covariance. Analyses were performed on an intention-to-treat basis. SPSS for Windows (version 17.0) was used to perform all statistical procedures.

## Results

### Patient characteristics

During the recruitment period we selected 537 patients, 212 of whom (40%) enrolled in the study; 151 (28%) refused to be contacted by the researcher or refused to participate in the study after the explanation of the research goals, and 174 (32%) could not be contacted after several unsuccessful attempts.

Table 1 shows the characteristics of patients randomised to the CCP, PACP and control conditions. Table 2 presents previous admissions and outpatient emergency visits.

**Table 1:** Baseline demographic and clinical characteristics of participant groups.

	PACP (n = 69)	CCP (n = 70)	Control group (n = 73)
Gender (%) male	50 (72.5)	46 (65.7)	49 (67.1)
Age (SD)	40.3 (10.9)	40.6 (11.6)	39.4 (11.6)
Ethnicity (%) Dutch	43 (62.3)	42 (60.0)	46 (63.0)
Diagnosis (%) Psychotic disorder	53 (76.8)	45 (64.3)	56 (76.7)
HoNOS (range)	11 (2–25)	11 (3–24)	10 (1–23)
Behaviour	2 (0–6)	1 (0–6)	1 (0–5)
Impairment	2 (0–5)	2 (0–6)	2 (0–6)
Symptoms	3 (0–9)	4 (0–9)	3,5 (0–9)
social problems	4 (0–10)	3 (0–9)	3 (0–9)

**Table 2:** Previous admissions and outpatient emergency visits.

Previous admissions and outpatient emergency visits			
No (%) of patients admitted	43 (62.3)	40 (57.1)	51 (69.9)
No (%) of patients with an emergency admission	13 (18.8)	12 (17.1)	18 (24.7)
No (%) of patients admitted under a court order	11 (15.9)	12 (17.1)	18 (24.7)
No (%) of patients who made one or more emergency outpatient visit	45 (65.2)	41 (58.6)	41 (56.2)

**Figure 1:**
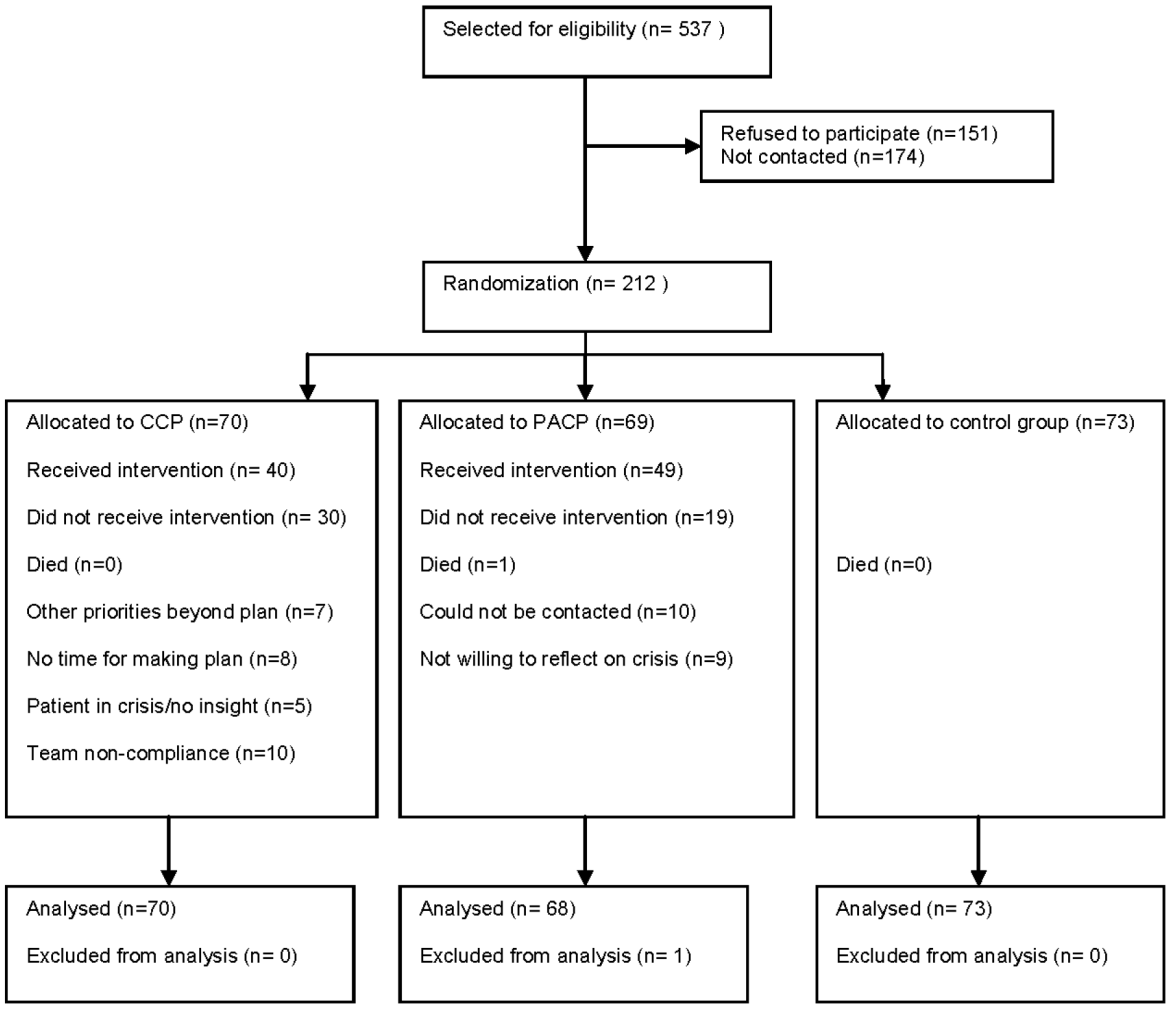
Participant flow chart.

For a flowchart of the study, see figure 1. Seventy percent of the patients (49/69) completed the PACP and 57% (40/70) completed the CCP. There was no drop out in the control condition from the study. The completion percentages in the two conditions were not significantly different. There were also no significant differences between the PACP and CCP completers and non-completers with respect to age, sex, diagnosis, ethnicity, education and marital status. The total duration of face-to-face contacts needed to draw up a crisis plan differed significantly between the PACP condition (Median = 120 minutes) and the CCP condition (Median = 180 minutes; Mann-Whitney U = 429,5; p = 0.00; r = −.36).

### Hospital admissions and outpatient emergency visits

Table 3 presents the numbers and percentages of patients who were admitted to hospital and who had emergency visits at follow up. Although not statistically significant, the percentages of overall admissions, emergency admissions and outpatient emergency visits were lower in both or either the PACP and CCP conditions compared to the control condition. For those admitted (N = 90), the number of bed days did not differ significantly between the three conditions (Kruskal-Wallis test, Chi-2 (2) = 2,1; p = 0.35). In the intervention conditions, the percentages of patients admitted voluntarily were higher, but not statistically significant, than in the control condition. Between the three conditions, the percentages of court-ordered admissions differed significantly, the percentages of patients in the PACP and CCP conditions being smaller than the percentage in the control condition. Table 4 shows that independently of the intervention condition, age and previous admission affect the chance of being voluntary hospitalised in the follow-up period. Controlling for confounders, patients in the CCP condition were *less* likely to be admitted under a court order than those in the control condition.

**Table 3:** Hospital admission and emergency visits at follow up.

	Intervention		Control Group (n = 73)	Chi^2^ –test**	Cramer's V
	PACP group (n = 69)	CCP group (n = 70)			
No (%) patients admitted	33 (47.8%)	24 (34.3%)	33 (45.2%)	0.3	0.34
No (%) patients admitted voluntarily	16 (23.2%)	14 (20.0%)	12 (16.4%)	1.0	0.07
No (%) patients with emergency admission	12 (17.4%)	7 (10.0%)	14 (19.2%)	1.1	0.07
No (%) patients admitted under court order	11 (15.9%)	7 (10.0%)	19 (26.0%)	5.7*	0.16
No (%) patients with emergency visits	22 (31.9%)	22 (31.4%)	25 (34.2%)	0.2	0.03

* P<0.05; df = 1.

** Chi^2^ test compares the intervention (PAPC+CCP) and the control group.

**Table 4:** Logistic regression results of admission at follow- up (court-ordered admission as reference).

	B (SE)	OR	95% CI for OR	P value
(Intercept)	1.421 (0.503)			
PAPC group	0.582 (0.416)	1.79	0.79 to 4.04	0.16
CCP group	0.960 (0.468)	2.61	1.04 to 6.54	0.04
Control group	0	1		
Male	−0.329 (0.428)	0.72	0.31 to 1.67	0.44
Age^1^	0.058 (0.018)	1.06	1.02 to 1.10	0.00
HoNOS^1^	−0.044 (0.036)	0.96	0.89 to 1.03	0.22
Dutch (versus immigrants)	−0.710 (0.386)	0.49	0.23 to 1.05	0.07
Not admitted before baseline	1.350 (0.477)	3.86	1.51 to 9.83	0.01
Bipolar disorder (versus psychotic disorder	0.788 (0.501)	2.20	0.82 to 5.88	0.12

Mc Fadden R^2 = ^ = 0.17, Model Chi^2^ = 40.5, df = 8, p = 0.00.

1 Grand mean centred.

### Effects on service engagement, social support, insight and the quality of the therapeutic alliance

There were no significant condition by time interactions between the interventions and the control condition: service engagement (F(2,381) = 0.27; p = 0.76); social support (F(2,532) = 2.1; p = 0.12); insight (F(2,547) = 1.9; p = 0.16); and working alliance (patient version: F(2,497) = 0.24; p = 0.78; therapist version: F(2,526) = 0.6; p = 0.58).

## Discussion

This randomized controlled trial showed that two types of plans did not significantly reduce overall admissions, voluntary admissions, emergency admissions, or outpatient emergency visits. Although not significant, there were fewer involuntary admissions and more voluntary admissions in the intervention conditions than in the control group. Crisis plans did have a significant effect on planned court-ordered admissions, especially when they had been composed together with the clinician. Independently of this effect, older participants who had not been admitted to psychiatric hospital before the study were less likely to be admitted under a court order. We did not find evidence for the associations between the effect of the crisis plans on court ordered admissions with service engagement, social support, insight and working alliance.

### Comparison with other studies

A systematic review identified only two studies on the effects of advance statements [21]. Recently, a third study was published [5]. The first of these, by Henderson et al. [4], found an effect of a joint crisis plan on the use of the Mental Health Act. In this study, the plan was developed together with the outpatient clinician, as was done in the CCP condition in our study. It may be that the involvement of the outpatient clinician is important for the effectiveness of the crisis plan. In the Henderson's study however, the intervention meeting was facilitated by an independent psychiatrist, what may have contributed to a better quality of the plan. Thornicroft et al. [5] re-examined the effect of a joint crisis plan made in the same fashion as described by Henderson et al [4] but on a larger scale using a multicentre design. Unfortunately they could not replicate the beneficial effect of a joint crisis plan on the use of the Mental Health Act. The authors suggest that the absence of a significant effect may be partially attributed to the insufficient implementation of the joint crisis plan at certain study sites. Finally, in the study by Papageorgiou et al. [6], patients wrote seven statements on their future preferences for treatment during their hospital stay, without any involvement of their outpatient clinicians, what may have disadvantaged the effectiveness of the statement.

### Limitations

This study had some limitations. Firstly, the DSM-IV diagnoses were not assessed by means of a structured diagnostic interview, making them less reliable; however such a diagnosis was of limited importance to the present study. Secondly, fewer patients were admitted than expected, what resulted in a lower statistical power to detect effects on the number of admissions. Thirdly, the generalisability of our results may have been limited because 60% of the eligible patients did not want to participate in the study. This refusal rate corresponded with that in the study by Henderson et al. [4], who reported a non-response of 64%; in the study by Papagourgiou et al. [6], the refusal rate was 30%. Fourthly, we did not have information on the manner in which the crisis plans were used in actual crisis situations. It may be that they were insufficiently used in clinical practice. Finally, another limitation is the high percentage of patients who did not complete the crisis plan: 30% in the PACP group and 43% in the CCP condition, which both contrast with the lower drop-out rate of 19% in the Henderson's study. Papageorgiou's study reported no explicit drop-out rate. Our drop out rate was nonetheless consistent with that in another study on facilitating the completion of psychiatric advance directives, in which 39% of participants did not complete such document [22]. In line with the intention to treat principle, effects of completers as well as non-completers were analysed together. Smaller numbers of admissions than anticipated, and fewer completers in the intervention condition, may have resulted in overall lower effects of the intervention. The study was underpowered to detect small beneficial effects of joint crisis plans.

### Clinical implications

Our study yielded three important results. Firstly, fewer patients were involuntarily admitted under a court order. Secondly, because a greater reduction in court-ordered admissions was found in the CCP than the PACP, it might be better to document a crisis plan together with the clinician than with a patient advocate. Thirdly, as we found no change in patient characteristics (see methods section), it is not clear which factors are associated with the reduction of court-ordered admissions. Therefore, we can only speculate on explanations for this result. It may be that the process of making a crisis plan by the patient and his or her clinician helps the clinician to feel more certain about what to do in times of a crisis situation, thereby reducing the need for court-ordered admissions, and causing a shift towards voluntary admissions. In other words, clinicians who have documented a crisis plan together with their patients may be better at risk assessment, and may therefore intervene earlier in order to prevent dangerous situations such as the self-neglect and social breakdown [23], [24].

In conclusion, our finding that a crisis plan could reduce court-ordered admissions may support the mental-health service policy of making advance statements a structural part of the treatment plans. However, experiences during this study showed that the participant clinicians needed intensive monitoring by the researcher. This suggests that the implementation of a crisis plan in the mental health system requires additional supervision.

Future research should replicate the results of this study and then focus on working mechanisms, cost-effectiveness of crisis plans and evaluate whether the instructions in the plans were followed during a particular crisis situation.

## Supporting Information

Protocol S1 **Trial Protocol.**
(DOC)

Protocol S2 **Trial Protocol.**
(DOC)

Checklist S1 **CONSORT Checklist.**
(DOC)

## References

[1] Swartz MS, Swanson JW, Hannon MJ (2003) Does fear of coercion keep people away from mental health treatment? Evidence from a survey of persons with schizophrenia and mental health professionals. Behav Sci Law 21: 459–472. 12898502 10.1002/bsl.539

[2] van der Gaag M (2002) Schizofrenie en posttraumatische stress-stoornis. Dth 22: 15–27.

[3] Salize HJ, Dressing H (2004) Epidemiology of involuntary placement of mentally ill people across the European Union. Br J Psychiatry 184: 163–8. 14754830 10.1192/bjp.184.2.163

[4] Henderson C, Flood C, Leese M, Thornicroft G, Sutherby K, et al (2004) Effect of joint crisis plans on use of compulsory treatment in psychiatry: single blind randomized controlled trail. BMJ 329: 136–138. 15240438 10.1136/bmj.38155.585046.63 PMC478218

[5] Thornicroft G, Farrelly S, Szmukler G, Birchwood M, Waheed W, et al (2013) Clinical outcomes of joint crisis plans to reduce compulsory treatment for people with psychosis: a randomised controlled trial. Lancet 381: 1634–1641. 23537606 10.1016/S0140-6736(13)60105-1

[6] Papageorgiou A, King M, Janmohamed A, Davidson O, Dawson J (2002) Advance directives for patients compulsorily admitted to hospital with serious mental illness. Br J Psychiatry 181: 513–519. 12456522 10.1192/bjp.181.6.513

[7] Srebnik DS, La Fond JQ (1999) Advance directives for mental health treatment. Psychiatr Serv 50: 919–925. 10402612 10.1176/ps.50.7.919

[8] Ruchlewska A, Mulder CL, Smulders R, Roosenschoon BJ, Koopmans G, et al (2009) The effects of crisis plans for patients with psychotic and bipolar disorders: a randomized controlled trial. BMC Psychiatry 9: 41. 19589145 10.1186/1471-244X-9-41 PMC2716324

[9] Henderson C, Swanson JW, Szmukler G, Thornicroft G, Zinkler M (2008) A typology of advance statements in mental health care. Psychiatr Serv 59: 63–71. 18182541 10.1176/ps.2008.59.1.63

[10] Wierdsma AI, Sytema S, van Os JJ, Mulder CL (2008) Case registers in psychiatry: do they still have a role for research and service monitoring? Curr Opin Psychiatry 21: 379–84. 18520743 10.1097/YCO.0b013e328304d99b

[11] Wing JK, Beevor AS, Curtis RH, Park SB, Hadden S, et al (1998) Health of the Nation Outcome Scale (HONOS). Research and development. Br J Psychiatry 172: 11–18. 9534825 10.1192/bjp.172.1.11

[12] Mulder CL, Staring ABP, Loos J, Buwalda VJA, Kuijpers D, et al (2004) De Health of the Nations Outcome Scales (HoNOS) als instrument voor ‘routine outcome assessment’. Tijd v Psychiatr 46: 273–285.

[13] Tait L, Birchwood M, Trower P (2002) A new scale (SES) to measure engagement with community mental health services. J Ment Health 11: 191–198. 21208145 10.1080/09638230020023570-2

[14] Wiznitzer M, Verhulst FC, van den Brink W, Koeter M, van der Ende, et al (1992) Detecting psychopathology in young adults: the Young Adult Self Report, the General Health Questionnaire and the Symptom Checklist as screening instruments. Acta Psychiatr Scand 86: 32–37. 1414396 10.1111/j.1600-0447.1992.tb03221.x

[15] Birchwood M, Smith J, Drury V, Healy J, Macmillan F, et al (1994) A self-report Insight Scale for psychosis: reliability, validity and sensitivity to change. Acta Psychiatr Scand 89: 62–67. 7908156 10.1111/j.1600-0447.1994.tb01487.x

[16] Horvath AO, Greenberg LS (1989) Development and validation of the Working Alliance Inventory. J Couns Psychology 36: 223–233.

[17] Vervaeke GAC, Vertommen H (1996) De werkalliantievragenlijst (WAV). Gedragstherapie 2: 139–144.

[18] Sutherby K, Szmukler GI, Halpern A, Alexander M, Thornicroft G, et al (1999) A study of “crisis cards” in a community psychiatric service. Acta Psychiatr Scand 100: 56–61. 10442440 10.1111/j.1600-0447.1999.tb10914.x

[19] Mulder CL, Wierdsma AI (2002) Voor wie is de acute dienst? Verschillen tussen eenmalige en frequente gebruikers. Tijd v Psychiatr 44: 523–531.

[20] Cohen J (1988) Statistical power analysis for the behavioral sciences (2nd edition). Hillsdale, NJ: Erlbaum.

[21] Campbell LA, Kisely SR (2009) Advance Treatment Directives for People with Severe Mental Illness. Cochrane Database Syst Rev 21: CD005963. 10.1002/14651858.CD005963.pub2 PMC4161493 19160260

[22] Swanson JW, Swarzt MS, Elbogen EB, van Dorn RA, Ferron J, et al (2006) Facilitated psychiatric advance directives: a randomized trial of an intervention to foster advance treatment planning among persons with severe mental illness. Am J Psychiatry 163: 1943–1951. 17074946 10.1176/appi.ajp.163.11.1943 PMC3747558

[23] Mulder CL, Tielens JAE (2008) Opvattingen over maatschappelijke teloorgang en zelfverwaarlozing beïnvloeden dwangopname. Tijd v Psychiatr 50: 229–233. 18491427

[24] Mulder CL, Uitenbroek D, Broer J, Lendemeijer B, van Veldhuizen JR, et al (2008) Changing patterns in emergency involuntary admissions in the Netherlands in the period 2000–2004. Int J Law Psychiatry 31: 331–336. 18667238 10.1016/j.ijlp.2008.06.007

