# Supplementary material for: Effect of Crisis Plans on Admissions and Emergency Visits: A Randomized Controlled Trial
Source: PLoS One. 2014 Mar 19;9(3):e91882. doi: 10.1371/journal.pone.0091882 (PMC3960137; doi:10.1371/journal.pone.0091882)
Supplement: Protocol S2 — Trial Protocol. (DOC) [file pone.0091882.s002.doc]

Protocol S2

###### Onderzoeksprotocol

**Crisiskaartonderzoek**

versie maart 2007

Effecten crisiskaart met of zonder inschakelen van een crisiskaartconsulent op het aantal crisiscontacten en (gedwongen) opnames

Drs. A. Ruchlewska

Prof. dr. C.L . Mulder

Drs. R. Smulders

Drs. B.J. Roosenschoon

#### Projectgroep

Projectleider Prof. dr. C.L Mulder

psychiater / onderzoeker

[niels.clmulder@wxs.nl](mailto:niels.clmulder@wxs.nl)

06-27034001

Hoofdonderzoeker Drs. A. Ruchlewska

psycholoog / promovendus

a.ruchlewska@bavo-europoort.nl

010-4363844

Begeleider Drs. R. Smulders

klinisch psycholoog / stafmedewerker

r.smulders@basisberaad.nl

010-4665962

Begeleider Drs. B.J. Roosenschoon

hoofd onderzoek / psycholoog

B.Roosenschoon@bavo-europoort.nl

010-4363844

Bestuurlijk M. Verschuure

verantwoordelijke directeur divisie Regionale Ketenzorg

010-4536789

Onafhankelijke arts Drs. M. de Pater

Psychiater

[M.depater@bavo-europoort.nl](mailto:M.depater@bavo-europoort.nl)

010- 4769821

Verrichter BavoEuropoort

##### Samenvatting

Deze studie betreft een gerandomiseerd onderzoek naar het effect van de crisiskaart met of zonder het inschakelen van een crisiskaartconsulent op het aantal crisissen en (gedwongen) opnames. Het onderzoek is van belang, ten eerste omdat crisissen en (gedwongen) opnames een grote impact hebben. Ten tweede omdat de aantallen crisissen en (gedwongen) opnames de laatste jaren toenemen. Ten derde omdat er een tekort bestaat aan werkzame interventies om crisissen en (gedwongen) opnames te voorkomen. Tenslotte is het onderzoek van belang omdat er twijfels zijn over de effectiviteit van de crisishulpverlening.

De effectiviteit van de crisishulpverlening kan mogelijk worden verbeterd door het opstellen van een crisiskaart en daarbij inschakelen van een onafhankelijke consulent. De crisiskaart is een combinatie van een crisisplan en samenvatting ervan op een soort bankpasje. De crisiskaart is bedoeld om signalen van dreigende crisissen tijdig te herkennen, en vervolgens te kunnen handelen volgens de afspraken die in het crisisplan zijn vastgelegd.

Uit eerder onderzoek zijn er aanwijzingen dat met de crisiskaart crisissen en (gedwongen) opnames kunnen worden voorkomen. Het is de vraag op welke manier een crisiskaart het beste kan worden opgesteld.

In het voorliggende onderzoek worden twee mogelijkheden onderzocht: (1) de crisiskaart opgesteld samen met een onafhankelijke crisiskaartconsulent en (2) de crisiskaart opgesteld samen met de behandelaar, zonder een consulent.

De achtergrond van het vergelijken van deze twee interventies is dat het mogelijk effectiever is om de crisiskaart op te stellen samen met een onafhankelijke consulent dan met de behandelaar. Met behulp van een onafhankelijke partij is de patiënt mogelijk vrijer om te bespreken wat hij/zij wel of niet wil tijdens een crisis. Een nadeel is dat het tijd kost en een extra schakel betekent bij het opstellen van een crisisplan. Dit kan drempelverhogend werken voor het opstellen van een crisiskaart. In het voorliggende onderzoek willen we daarom onderzoeken of het inschakelen van een crisiskaartconsulent de vraaggerichtheid en het aantal crisissen en (gedwongen) opnames positief beïnvloedt.

Het onderzoek heeft betrekking op patiënten met psychotische of bipolaire stoornissen die één of meer crisiscontacten of opnames in de afgelopen twee jaar hebben gehad. Zij worden geworven door behandelaars uit teams voor patiënten met ernstige psychiatrische stoornissen.

Er zullen drie groepen met ieder 80 patiënten worden vergeleken: groep 1: crisiskaart opgesteld door de patiënt samen met een crisiskaarconsulent; groep 2: crisiskaart opgesteld door de patiënt samen met de behandelaar, zonder consulent en groep 3: controlegroep zonder crisiskaart.

Primaire effectmaten zijn het aantal crisiscontacten en (gedwongen) opnames en de opnameduur. Secundaire effectmaten zijn psychisch en sociaal functioneren. Daarnaast zal explorerend worden onderzocht wat de werkzame factoren zijn die bijdragen aan een eventueel effect van de crisiskaart. De follow-up periode is 18 maanden.

[1 Achtergrond 6](#__RefHeading___Toc161139774)

[1.1 Probleemstelling 6](#__RefHeading___Toc161139775)

[1.2 Relevantie 7](#__RefHeading___Toc161139776)

[1.2.1 Versterking vraaggerichtheid 7](#__RefHeading___Toc161139777)

[1.2.2 Algemene relevantie 8](#__RefHeading___Toc161139778)

[1.3 Kennisoverdracht 8](#__RefHeading___Toc161139779)

[2 Doelstelling 9](#__RefHeading___Toc161139780)

[3 Vraagstelling 9](#__RefHeading___Toc161139781)

[4 Studie design 9](#__RefHeading___Toc161139782)

[5 Onderzoekspopulatie 10](#__RefHeading___Toc161139783)

[5.1 Patiënten 10](#__RefHeading___Toc161139784)

[5.2 Inclusie criteria 10](#__RefHeading___Toc161139785)

[5.3 Exclusie criteria 10](#__RefHeading___Toc161139786)

[5.4 Steekproefgrootte 10](#__RefHeading___Toc161139787)

[6 Onderzoeksinterventie 10](#__RefHeading___Toc161139788)

[7 Onderzoeksgroepen 11](#__RefHeading___Toc161139789)

[8 Methode 12](#__RefHeading___Toc161139790)

[8.1 Uitkomstmaten 12](#__RefHeading___Toc161139791)

[8.1.1 Primaire uitkomstmaten 12](#__RefHeading___Toc161139792)

[8.1.2 Secundaire uitkomstmaten 12](#__RefHeading___Toc161139793)

[8.1.3 Werkzame factoren 13](#__RefHeading___Toc161139794)

[8.2 De route van het onderzoek 13](#__RefHeading___Toc161139795)

[8.2.1 Selectie van de patiënten 13](#__RefHeading___Toc161139796)

[8.2.2 Training van de behandelaars 13](#__RefHeading___Toc161139797)

[8.2.3 Werving van de patiënten 14](#__RefHeading___Toc161139798)

[8.3 De procedure 14](#__RefHeading___Toc161139799)

[8.3.1 Onderzoeksgegevens 14](#__RefHeading___Toc161139800)

[8.3.3 Tijdschema van de studie 15](#__RefHeading___Toc161139801)

[8.4 Participerende locaties 15](#__RefHeading___Toc161139802)

[9 Statistiek 15](#__RefHeading___Toc161139803)

[10 Haalbaarheid van de studie 15](#__RefHeading___Toc161139804)

[10.1 Tijdspad 16](#__RefHeading___Toc161139805)

[11 Ethische overwegingen 16](#__RefHeading___Toc161139806)

[11.1 Werving en informed consent 16](#__RefHeading___Toc161139807)

[11.2 Randomisatie en weigering 16](#__RefHeading___Toc161139808)

[Referenties 17](#__RefHeading___Toc161139809)

[Bijlage 1 Stroomschema onderzoeksprotocol 20](#__RefHeading___Toc161139810)

[Bijlage 2 Stroomschema randomisatie 21](#__RefHeading___Toc161139811)

[Bijlage 3 Overzicht instrumentarium 22](#__RefHeading___Toc161139812)

[Bijlage 4 Onderzoekscontact met patiënt per meetmoment 23](#__RefHeading___Toc161139813)

[Bijlage 5 Theoretisch model 24](#__RefHeading___Toc161139814)

# 1 Achtergrond

## 1.1 Probleemstelling

Crisissen en (gedwongen) opnames hebben een grote impact op de patiënt en diens omgeving (Swartz ea 2003). Ongeveer 50% van de patiënten ervaart gedwongen opnames als traumatiserend (van der Gaag 2002). Vanaf 2000 - 2003 zijn het aantal ambulante crisiscontacten met 106% gestegen (van 73.177 tot 151.096 contacten; info GGZ-Nederland), het aantal opnames met 162% en het aantal inbewaringstellingen met 17% (van 6346 tot 7450; gegevens IGZ). Deze stijgingen vonden vooral plaats onder patiënten die al bekend zijn in de GGZ (Mulder ea 2006).

De oorzaken van deze stijgingen zijn divers: meer ambulant gaan werken van GGZ-instellingen, een tendens om eerder in te grijpen bij crisissituaties, en een toenemende neiging om dakloze patiënten van straat te halen (Mulder ea 2006). Daarnaast kunnen de stijgingen een signaal zijn van tekorten in het ambulante aanbod en een gebrek aan vroegtijdige signalering van aankomende crisissen (Basisberaad 2004).

Een relatief eenvoudig instrument zoals de crisiskaart (een vorm van crisisprotocol) blijkt in de praktijk nog vaak ongebruikt.

Effect-onderzoek naar interventies om crisissen en (gedwongen) opnames te voorkomen is schaars (Srebnik ea 2005).

Twee onderzoeken bestudeerden de effecten van instrumenten zoals de crisiskaart

('advanced directives'). Uit één studie bleek dat 13% van de patiënten met wie een 'joint crisis plan' was opgesteld gedwongen werden opgenomen, tegenover 27% van de patiënten zonder plan (een significant verschil; Henderson ea 2004). Dit plan werd door de patiënt samen met diens ambulante behandelaar opgesteld. De patiënt kreeg hierbij ondersteuning van de lokale patiëntenvereniging en van een belangrijke ander uit zijn omgeving.

Een tweede onderzoek vond geen effecten van een ander soort crisisprotocol (Papageorgiou ea 2002).

Dit protocol bestond uit een soort boekje (“booklet “). Patiënten schreven tijdens een gedwongen opname zelfstandig een zevental punten in het boekje die van belang waren voor een toekomstige crisis. Dit boekje werd in het dossier bewaard. Mogelijk zijn de patiënten en de behandelaars zich onvoldoende bewust geweest van de aanwezigheid van het boekje na het ontslag. Dit heeft er wellicht toe geleid dat de patiënten en de behandelaars het boekje niet geraadpleegd hebben tijdens een crisis. Het 'joint crisis plan' lijkt een krachtiger instrument dan het boekje vanwege het inschakelen van de behandelaar, iemand van de patiëntenvereniging en een voor de patiënt belangrijke ander.

Factoren die de (eventuele) werkzaamheid van een crisisprotocol bepalen zijn onbekend (Sutherby ea 1999). Misschien leidt een crisisprotocol tot een toegenomen gevoel van veiligheid en controle (self-efficacy) en een vermindering van ervaren stress; misschien herkennen patiënten met een crisisprotocol de signalen van een aankomende crisis eerder en zoeken ze eerder hulp. Ook kan het proces van het maken van een crisisprotocol een therapeutische werking hebben, bijvoorbeeld door het meer accepteren van de ziekte, het verbeteren van de relatie met de behandelaar, of door een toegenomen mondigheid van de patiënt (Thomas 2003; Basisberaad 2004). Volgens de patiëntenvereniging werkt het opstellen van een crisiskaart samen met een onafhankelijk consulent versterkend bij het tot stand komen van bovengenoemde werkzame ingrediënten: de patiënt kan zich vrijer voelen om te zeggen wat hij wel of niet wil bij een toekomstige crisis en hij kan eventuele eerdere negatieve ervaringen met de crisishulpverlening met de crisiskaartconsulent bespreken (Basisberaad 2004).

Er zijn geen empirische gegevens over de mogelijk werkzame factoren en in de voorliggende studie zal dit onderzocht worden.

In Nederland bestaan verschillende soorten crisisprotocollen, waaronder de crisiskaart. De crisiskaart is ontwikkeld door de patiëntenbelangenorganisatie en voortgekomen uit een gebrek aan samenwerking tussen patiënten en de crisishulpverlening (zie relevantie). De crisiskaart heeft twee doelen: het voorkomen van crisissen en (gedwongen) opnames en het beschrijven hoe te handelen bij een (dreigende) crisis.

De patiënt stelt de crisiskaart op samen met een onafhankelijke consulent (vertegenwoordiger van een patiëntenbelangenorganisatie). De behandelaar ziet het uiteindelijke resultaat en ondertekent mede de crisiskaart. De crisiskaartconsulent wordt gezien als een belangrijke succesfactor voor de werking van crisiskaart. Een nadeel is dat het tijd kost en een extra schakel betekent bij het opstellen van een crisisprotocol. Tot nu toe is het aantal crisiskaarten in Nederland beperkt.

Conclusie: Het aantal crisiscontacten en (gedwongen) opnames neemt toe in Nederland. Er is behoefte aan werkzame methodes om deze tendens te keren. De crisiskaart is mogelijk een geschikte methode. In de voorliggende studie willen we daarom onderzoeken of een crisiskaart opgesteld met of zonder crisiskaartconsulent crisissen en (gedwongen) opnames kan voorkomen, en wat de eventuele werkzame factoren zijn.

## 1.2 Relevantie

### 1.2.1 Versterking vraaggerichtheid

Het gaat hier om het uittesten van een vorm van crisisprotocol die patiënten in staat stelt om als goed geïnformeerde consument te participeren in het eigen hulpverleningsproces. De vorm van crisisprotocol die hier gekozen is, is de crisiskaart. Deze is ontwikkeld op initiatief van het Amsterdams Patiënten- en Consumentenplatform (APCP) in 1999, naar aanleiding van ontevredenheid met de behandeling van patiënten tijdens crisissituaties.

In de crisiskaart is vastgelegd hoe patiënten tijdens een crisis behandeld willen worden. De crisiskaart kan voorkomen dat er beslissingen worden genomen tijdens een crisis die indruisen tegen de wensen van de patiënt. Daarnaast heeft de crisiskaart tot doel om crisissen te voorkomen door het vroeg inspelen op signalen die wijzen op een aankomende crisis (bijvoorbeeld wanneer de patiënt merkt dat hij slechter slaapt of weer stemmen begint te horen).

Het is de vraag of het opstellen van een crisiskaart samen met de crisiskaartconsulent (in plaats van alleen met de behandelaar) het effect van de crisiskaart en de vraaggerichtheid positief beïnvloedt.

Dit zou kunnen omdat de patiënt zich in aanwezigheid van een crisiskaartconsulent mogelijk vrijer voelt om precies aan te geven wat hij/zij wil tijdens een crisis, dan wanneer de patiënt en de behandelaar samen de crisiskaart opstellen (Basisberaad 2004). Dit kan leiden tot een crisiskaart waarin de wensen van de patiënt beter beschreven staan. Hierdoor kan patiënt zich beter voorbereid voelen op een crisis waardoor hij minder angstig is, minder snel in crisis raakt of signalen eerder herkent en hulp in kan roepen. Hierdoor kunnen (gedwongen) opnames worden voorkomen.

Het omgekeerde kan echter ook gebeuren, aangezien de crisiskaartconsulent een onbekende is voor de patiënt en hij /zij de situatie van de patiënt niet kent. Wanneer de crisiskaart is opgesteld met een crisiskaartconsulent, en niet alleen met de behandelaar, kan het zo zijn dat de crisiskaart in de praktijk minder goed bruikbaar is. Zo kunnen er bijvoorbeeld afspraken instaan die in de praktijk moeilijk uitvoerbaar zijn.

Wanneer blijkt dat één van beide interventies (of beide) effectief is, kan deze worden geïmplementeerd in de dagelijkse patiëntenzorg voor de boven genoemde patiëntengroepen.

### 1.2.2 Algemene relevantie

Zoals in de probleemstelling is aangegeven, heeft een crisis en een (gedwongen) opname een grote invloed heeft op de patiënt en diens omgeving. Gedwongen opnames kunnen als een trauma beleefd worden en leiden tot een afkeer van de GGZ (Swartz ea 2003). Daarnaast is het aantal crisiscontacten en (gedwongen) opnames de laatste jaren sterk gestegen. Voorlopig lijkt er geen einde te komen aan deze stijging (Mulder ea 2006). Om deze redenen is het van groot belang om interventies te ontwikkelen die effectief zijn in het voorkomen van crisissen en (gedwongen) opnames.

Verder is het belangrijk om inzicht te krijgen in de werkzame factoren die het eventuele effect van een crisisplan bepalen: verhogen van een gevoel van veiligheid en controle (self-efficacy), vermindering van stress, eerder en effectiever ingrijpen in geval van een (dreigende) crisis, verbetering van de therapeutische alliantie, therapietrouw, of meer acceptatie van de ziekte. Inzicht in deze factoren kan helpen bij het verbeteren van crisisplannen en het ontwikkelen van nieuwe interventies ter voorkoming van crisissen en (gedwongen) opnames. Tenslotte past het onderzoek naar de effecten van de crisiskaart in een breder kader naar de mogelijkheden tot het ontwikkelen van vormen van zelfbinding in de psychiatrie.

Er loopt voor zover ons bekend geen vergelijkbaar onderzoek op dit gebied. Wel wordt momenteel een kwalitatief onderzoek gedaan naar de ervaringen van patiënten met de crisiskaart in samenwerking met de Landelijke Werkgroep Crisiskaart. Het voorliggende onderzoek is vooral kwantitatief van aard, en onderzoekt (ook in samenwerking met de Landelijke Werkgroep) de effecten van de crisiskaart.

## 1.3 Kennisoverdracht

Het voorgestelde onderzoek levert inzicht op in de werkzaamheid van de crisiskaart en in de

meerwaarde van het inschakelen van een crisiskaartconsulent. De implementatie van het onderzoek zal leiden tot een sterke toename van de kennis over de crisiskaart onder de deelnemende locaties.

Deelname van de GGZ instelling, samen met de patiëntenbelangenorganisatie (Basisberaad) aan het onderzoek maakt dat kennis over de (effectiviteit van) de crisiskaart breed kan worden verspreid. Daarnaast zullen vanaf de start van het onderzoek tijdens (inter)nationale symposia en congressen referaten en posters over het onderzoek en de crisiskaart gehouden worden. De resultaten van het onderzoek zullen tevens worden gepubliceerd in algemene en wetenschappelijke nationale en internationale tijdschriften en in de vorm van een proefschrift.

De resultaten van het onderzoek zullen implicaties hebben voor de toepassing van crisisprotocollen. Kennisoverdracht zal plaatsvinden door de onderzoeker en de leden van de projectgroep. De leden van de projectgroep vormen een vertegenwoordiging van de instelling en de patiëntenbelangenvereniging. Deelname van de patiëntenbelangenorganisatie (het Basisberaad) aan het onderzoek maakt het mogelijk dat kennis over de effecten van de crisiskaart onder andere patiëntenbelangenorganisaties verspreid kan worden. Ook geeft het onderzoek een impuls aan gezamenlijke onderzoeksactiviteiten met de patiëntenbelangenorganisaties.

# 2 Doelstelling

Het doel van deze studie is om de effecten van de crisiskaart en de inschakeling van een onafhankelijke crisiskaartconsulent te onderzoeken. Daarnaast kan het inzicht in de mogelijke werking van de crisiskaart hulp bieden bij de verbetering van crisisprotocollen en bij het ontwikkelen van nieuwe interventies ter voorkoming van crisissen en (gedwongen) opnames.

# 3 Vraagstelling

De vraagstellingen van het onderzoek zijn als volgt:

(1) Wat zijn de effecten van de crisiskaart op het aantal crisiscontacten, (gedwongen) opnames en de opnameduur (los van of de crisiskaart is opgesteld met of zonder een crisiskaartconsulent)?

(2) Is het effect van de crisiskaart afhankelijk van het inschakelen van een crisiskaartconsulent?

(3) Wat is het effect van de crisiskaart (opgesteld met of zonder crisiskaartconsulent) op de vraaggerichtheid en het psychisch en sociaal functioneren?

(4) Wat zijn de eventuele werkzame factoren van een crisiskaart, met of zonder inschakelen van een crisiskaartconsulent?

De hypotheses zijn:

(1) Het bezit van een crisiskaart (los van of de crisiskaart is opgesteld met of zonder een crisiskaartconsulent) leidt tot een vermindering van het aantal crisiscontacten,

(gedwongen) opnames en opnameduur ten opzichte van het niet hebben van een crisiskaart.

(2) Het effect van de crisiskaart is groter wanneer een crisiskaartconsulent is ingeschakeld

(3) De crisiskaart (opgesteld met of zonder crisiskaartconsulent) heeft een positief effect op vraaggerichtheid het psychisch en sociaal functioneren

(4) Werkzame factoren voor de eventuele effecten van de crisiskaart (opgesteld met of zonder crisiskaartconsulent) zijn een toegenomen gevoel van controle, acceptatie van de ziekte, betere therapietrouw, verbeterde relatie met de behandelaar en toegenomen sociale steun.

# 4 Studie design

Deze studie betreft een gerandomiseerd onderzoek waarbij patiënten met toestemming worden toegewezen aan twee experimentele condities en een controlegroep:

- Groep 1. Crisiskaart opgesteld door de patiënt samen met een crisiskaartconsulent.

- Groep 2. Crisiskaart opgesteld door de patiënt samen met de behandelaar.

- Groep 3. Controlegroep zonder crisiskaart.

# 5 Onderzoekspopulatie

## 5.1 Patiënten

Het onderzoek vindt plaats bij patiënten met psychotische en bipolaire stoornissen die *'at risk'* zijn voor crisissen en (gedwongen) opnames.

## 5.2 Inclusie criteria

Inclusiecriteria zijn:

- De patiënt is momenteel in ambulante behandeling.

- Gedurende de afgelopen twee jaren heeft de patiënt één of meer crisiscontacten en/of één of meer (gedwongen) opnames gehad.

- Psychotische of bipolaire stoornis II.

Deze criteria zijn gekozen omdat deze patiënten een verhoogd risico lopen op een herhaald

crisiscontact of een heropname (Mulder ea 2006) en omdat bij deze groep patiënten een vorm van crisisprotocol effectief gebleken is (Henderson ea 2004).

## 5.3 Exclusie criteria

Exclusiecriteria zijn:

- Organisch psychosyndroom.

- Wilsonbekwaamheid ten aanzien van het geven van informed consent.

- Onvoldoende beheersing van de Nederlandse taal.

## 5.4 Steekproefgrootte

De steekproefgrootte is door middel van de power analyse bepaald.

De power analyse voor het aantonen van een verschil tussen wel of geen crisiskaart is gebaseerd op de resultaten van Henderson et al (2004). Uitgaande van een power van 0.90 (0.80 wordt in de regel als ondergrens genomen), en een alpha=0.05 vergt het aantonen van verschillen in opnamekans tussen de interventiegroepen en de controlegroep een steekproef van 70 personen per groep; we hebben 160 personen in de twee interventiegroepen en 80 personen in de controlegroep. We hebben geen data kunnen vinden voor een poweranalyse over de benodigde aantallen patiënten om een verschil tussen de beide interventies te kunnen aantonen. In het algemeen geldt, wanneer we uitgaan van een gemiddelde effectsize (0.6), alpha =0.05 en een power van 0.85, dat er minimaal 50 personen per conditie nodig zijn. Met 80 personen per interventiegroep is de power voldoende.

# 6 Onderzoeksinterventie

De interventie is de crisiskaart.

De crisiskaart is bedoeld voor patiënten met ernstige psychiatrische stoornissen die een verhoogd risico lopen om in crisis te raken en (gedwongen) opgenomen te worden. De crisiskaart is gebaseerd op een maximaal vier pagina's tellend crisisplan.

De inhoud van het crisisplan (zie onder) staat samengevat op een klein uitvouwbaar kaartje van het formaat bankpas: de crisiskaart. Wanneer wij hieronder crisiskaart schrijven, bedoelen we hiermee het crisisplan inclusief de crisiskaart. Bij het opstellen van de crisiskaart zal zoveel mogelijk geprobeerd worden om een familielid, partner of vriend van de patiënt te betrekken; de vertrouwenspersoon (wordt geregistreerd).

Het opstellen van een crisiskaart gebeurt wanneer de patiënt niet in een crisissituatie verkeert en in staat is om samen met de crisiskaartconsulent en / of de behandelaar (zie onder) de inhoud van de crisiskaart te bepalen. Het opstellen van de crisiskaart neemt twee tot drie bijeenkomsten in beslag (de tijdsduur voor het maken van een crisiskaart wordt geregistreerd). De patiënt, eventueel de crisiskaartconsulent en de behandelaar (zie onder) ondertekenen de crisiskaart en passen de crisiskaart aan wanneer dit gewenst is.

Uiteindelijk komt de crisiskaart in het patiëntendossier. Ook komt de crisiskaart ter beschikking van de acute dienst (die kan buiten kantooruren in aanraking komen met de patiënt). Een kopie van de crisiskaart ligt op het crisiscentrum in Rotterdam of op de opnameafdeling, indien de patiënt eerder opgenomen geweest is. Hierdoor is de inhoud van de crisiskaart 7 maal 24 uur bereikbaar, zodat informatie uit de crisiskaart altijd toegankelijk is. De patiënt draagt de samenvatting van het plan op de crisiskaart altijd bij zich.

De volgende informatie komt in de crisiskaart:

- Algemene gegevens en gebruikte medicatie.

- Hoe is de patiënt buiten een crisis om, in zijn / haar normale doen.

- Hoe ziet een crisis van de patiënt er doorgaans uit: mogelijke aanleidingen en signalen en gedrag tijdens een crisis.

- Rol van de vertrouwenspersoon: bij de signalering, of bij het regelen van een opname of de

vertegenwoordiging van de patiënt wanneer deze niet meer wilsbekwaam is.

- Wensen en adviezen aan de hulpverlening (behandelaar, crisisdienst, opnamekliniek) en mogelijke anderen die betrokken zijn bij een crisis (bijvoorbeeld politie of ambulance personeel) ten aanzien van wat wel en niet te doen tijdens een crisis: welke medicatie wel en niet gebruiken (bijvoorbeeld in verband met ervaren bijwerkingen), hoe met de patiënt om te gaan (bijvoorbeeld niet aanraken) en praktische zaken (zoals de opvang van huisdieren).

- Wat de patiënt absoluut niet wil, bijvoorbeeld niet alleen gelaten worden, of bepaalde dingen niet zeggen.

- Gegevens betreffende de lichamelijke aandoeningen.

- Afspraken die gemaakt zijn met opname-instellingen.

Juridisch gezien geldt de crisiskaart deels als een (niet juridisch bindende) wilsverklaring en deels als een onderdeel van de behandelovereenkomst, waar behandelaars in het kader van de WGBO alleen op goede, professionele gronden van mogen afwijken.

# 7 Onderzoeksgroepen

Groep 1.

De patiënt stelt samen met de crisiskaartconsulent de crisiskaart op en beiden ondertekenen deze. De crisiskaartconsulent is in dienst van de patiëntenvereniging en kent de instellingen in de regio. Hij /zij is deskundig in het opstellen van een crisiskaart. De consulent is getraind in vraaggericht werken en stelt de wensen van de patiënt centraal (over hoe te handelen tijdens een crisis). Wanneer de crisiskaart gereed is, ondertekent ook de behandelaar de crisiskaart.

Groep 2.

De patiënt stelt de crisiskaart samen met de behandelaar op in het kader van de lopende behandeling en zij ondertekenen deze beiden. Behandelaars ontvangen hiervoor een eenmalige instructie van de crisiskaartconsulenten en schriftelijk materiaal over de inhoud van de crisiskaart. De crisiskaartconsulent wordt verder niet bij het opstellen van de crisiskaart betrokken.

Groep 3.

De controlegroep ontvangt de normale behandeling, zonder het opstellen van een crisiskaart. Wanneer patiënten uit de controlegroep eventueel toch vragen om het opstellen van een crisiskaart zal dit gebeuren. Dit zal apart geregistreerd worden. Deze patiënten blijven wel in de studie.

# 8 Methode

## 8.1 Uitkomstmaten

### 8.1.1 Primaire uitkomstmaten

Primaire uitkomstmaten betreffen het aantal crisiscontacten, het aantal (gedwongen = IBS) opnames en de opnameduur.

Een crisiscontact is gedefinieerd als een dringend (= de problematiek vereist een face to face hulpverleningscontact binnen 24 uur) en niet gepland (< 24 uur) of acuut face to face contact met een behandelaar. Dit wordt binnen instellingen apart geregistreerd. Crisiscontacten vinden meestal plaats met een behandelaar van de crisisdienst en kunnen binnen en buiten kantooruren plaatsvinden (wordt geregistreerd).

### 8.1.2 Secundaire uitkomstmaten

Secundaire effectmaten zijn vraaggerichtheid en psychisch en sociaal functioneren.

1. Vraaggerichtheid

(a) Binnen een maand na het opstellen van de crisiskaart zal met een aparte (te ontwikkelen) korte vragenlijst worden nagegaan in hoeverre de patiënt vindt dat zijn/haar eigen visie in het plan terecht is gekomen en in hoeverre de patiënt zich vrij voelde om te spreken over wat hij/zij wel of niet zou willen tijdens een crisis.

(b) Tevredenheid met behandeling: Hiervoor zal de Thermometer van GGZ-Nederland worden gebruikt; speciaal zal gekeken worden naar antwoorden op de drie items over inspraak in de behandeling.

(c) Tevens zal tijdens de follow-up geïnventariseerd worden of de crisiskaart gebruikt is, of men baat heeft gehad bij de crisiskaart (en zo ja hoe), en of de afspraken die op de crisiskaart zijn vastgelegd werkzaam zijn gebleken en zijn nagekomen. Hiervoor zal een aparte gestructureerde vragenlijst worden ontwikkeld.

2. Het effect op psychisch en sociaal functioneren. Hiervoor zal de Health of the Nation Outcome Scales gebruikt worden (HoNOS; 12 items; Wing ea 1998; Mulder ea 2002). De HoNOS is een beoordelingsschaal met twaalf items en geeft informatie over agressief gedrag, suicidaliteit, misbruik van middelen, cognitief functioneren, lichamelijke problemen, depressieve klachten, symptomen van wanen of hallucinaties, overige psychische klachten, woonsituatie, sociale contacten, ADL-functies, en problemen met de hulpverlening. Deze schaal is onderzocht voor Nederland en bleek betrouwbaar, valide en gevoelig voor verandering (Mulder ea 2002).

### 8.1.3 Werkzame factoren

De volgende gegevens over mogelijk werkzame factoren zullen worden verzameld:

(a) Gevoel van veiligheid en controle over (dreigende) crisis, timing bij inroepen hulp igv (dreigende) crisis: te ontwikkelen korte vragenlijst.

(b) Acceptatie van de psychiatrische ziekte: Ned. vertaling van de Recovery Style Questionnaire (RSQ, 39 items; Drayton ea 1998). Deze vragenlijst bepaalt in hoeverre de patiënt zijn psychiatrische aandoening accepteert ('integration') of verdringt ('sealing over'). De Engelse versie heeft goede psychometrische eigenschappen.

(c) Therapeutische relatie met de behandelaar: Ned. Vertaling van de Working Alliance Inventory (WAI, 36 items; Horvath ea 1989), in te vullen door respectievelijk de patiënt en zijn behandelaar.

(d) Therapietrouw: Ned. vertaling van de Service Engagement Scale (SERVES; 14 items; Tait ea 2002; 2003). Deze beoordelingsschaal bepaalt in hoeverre een patiënt medicatietrouw is, afspraken nakomt en geneigd is hulp in te roepen bij een (dreigende) crisis. De schaal wordt ingevuld door de onderzoeker op basis van een interview met de behandelaar. De Engelse versie heeft goede psychometrische eigenschappen.

(e) Sociale steun: Ned. vertaling van de Adult Self Report (ASR; 14 items; Achenbach 2003).

## 8.2 De route van het onderzoek

### 8.2.1 Selectie van de patiënten

Behandelaars uit teams voor patiënten met langdurige en ernstige psychiatrische stoornissen van de Bavo Europoort doen aan het onderzoek mee (ongeveer 2000 patiënten). Patiënten die voldoen aan de inclusiecriteria (verwachte n=750) zullen worden gevraagd om mee te doen aan het onderzoek. Eerder onderzoek liet zien (Henderson et al. 2004) dat ongeveer 50% van de patiënten bereid is om aan een dergelijke studie mee te doen (n= 375). In de praktijk blijkt de respons echter vaak tegen te vallen. Met deze aantallen denken we in ieder geval 240 patiënten te kunnen includeren.

### 8.2.2 Training van de behandelaars

Een maand voor het starten van de randomisatie krijgen de behandelaars tijdens een bijeenkomst voorlichting over de procedure van het maken van de crisiskaart.

Tijdens deze bijeenkomst zal de onderzoeker het onderzoek presenteren gevolgd door voorlichting over de crisiskaart door de crisiskaartconsulenten. De duur van deze bijeenkomst is ongeveer 3 uur. Vervolgens krijgen de behandelaars een protocol over de te volgen procedures.

### 8.2.3 Werving van de patiënten

De behandelaar selecteert uit zijn patiëntenbestand de patiënten die voldoen aan de in- en exclusiecriteria. Vervolgens ontvangen deze geselecteerde patiënten informatie en een informatiefolder over het onderzoek en wordt hen gevraagd of zij het goed vinden dat de onderzoeker contact met ze opneemt.

De onderzoeker neemt dan contact op met de patiënt, legt hem/haar het onderzoek nog een keer uit en vraagt om schriftelijke toestemming voor deelname aan het onderzoek. Er wordt hierbij genoemd dat de reden van een mogelijke weigering tot deelname niet hoeft te worden opgegeven. Bij weigering tot deelname aan het interventieonderzoek wordt gevraagd om diagnostische gegevens uit het dossier te mogen gebruiken, om de generaliseerbaarheid van de onderzoeksdeelname te kunnen bepalen. Wanneer een patiënt dit niet wil, worden deze gegevens niet gebruikt.

## 8.3 De procedure

### 8.3.1 Onderzoeksgegevens

Gegevens over de primaire, secundaire uitkomstmaten en de werkzame factoren zullen verzameld worden op baseline (voorafgaande aan de randomisatie) en na 9 en 18 maanden.

Tijdens het eerste contact (baseline) en nadat de patiënt schriftelijke toestemming heeft gegeven zullen de vragenlijsten en het interview worden afgenomen. Vervolgens vindt de randomisatie plaats.

Ieder meetmoment zal maximaal 60 minuten duren. Indien gewenst zal het interview op twee momenten plaatsvinden. De onderzoekscontacten zullen op de afdeling waar de patiënt behandeld wordt plaatsvinden.

Naast de eerder genoemde uitkomstmaten en werkzame factoren worden op baseline ook de volgende gegevens verzameld:

- Demografische gegevens: leeftijd, geslacht, woon- een leefsituatie, bron van inkomsten, opleiding, geboorteland patiënt, vader en moeder.

- Psychiatrische voorgeschiedenis: aantal crisiscontacten gedurende het afgelopen twee jaar en het aantal opnames, IBS-en en Rechterlijke Machtigingen (totaal en afgelopen twee jaar).

- Psychiatrische aandoening: DSM-IV-TR diagnostiek (vijf assen) volgens de behandelaar. Wij realiseren ons dat dit niet altijd betrouwbaar is, maar het is niet mogelijk om met de beschikbare middelen een gestandaardiseerd diagnostisch interview af te nemen.

- Verwachtingen van de crisiskaart: aan de patiënt en de behandelaar wordt gevraagd of men de crisiskaart een nuttig instrument vindt en of men verwacht er baat bij te hebben.

De demografische variabelen, psychiatrische voorgeschiedenis en de psychiatrische aandoening worden verzameld door de onderzoeker uit de administratie van de instelling (een crisiscontact kent een aparte code en tarief), aangevuld met gegevens uit het dossier. De onderzoeker kan niet blind zijn voor de (experimentele- of controle-) conditie van de patiënt, aangezien het crisisplan in het dossier wordt bewaard.

Tevens zal de voorgeschreven medicatie geregistreerd worden om hiermee rekening te kunnen houden bij de analyses.

Na ieder interview ontvangen patiënten het bedrag van €10, in totaal 4 keer, als blijk van waardering voor deelname aan het onderzoek.

De onderzoeksgegevens worden op een anonieme wijze verwerkt. Persoongegevens wordt een nummer toegekend. Het onderzoeksteam heeft toegang tot de sleutel van de code.

### 8.3.3 Tijdschema van de studie

Tijdschema van de studie (T = Tijdstip)

T = maand -1 Werving van de patiënten.

T = maand 0 Baseline meting, incl. de primaire en secundaire uitkomstmaten en de werkzame factoren.

T = maand 1 Randomisatie en vervolgens toewijzen naar 1 van de 3 groepen.

T = maand 1-3 Opstellen van de crisiskaart.

T = maand 4 Vastleggen vraaggerichtheid van de crisiskaart.

T = maand 9 Follow-up meting primaire en secundaire uitkomstmaten en werkzame factoren.

T = maand 18 Follow-up meting primaire en secundaire uitkomstmaten en werkzame factoren.

De geplande startdatum van de studie is 1 oktober 2007 en de verwachte einddatum is 1 februari 2010.

## 8.4 Participerende locaties

In totaal doen acht locaties van de divisie regionale ketenzorg van BavoEuropoort aan het onderzoek mee, met in totaal ongeveer 2000 patiënten, waarvan ongeveer de een derde aan

de inclusiecriteria voldoet.

# 9 Statistiek

Bij de randomisatie passen we de 'minimisation method' toe (Beller ea 2002). Dit is een dynamische random allocatie methode, met de mogelijkheid om te stratificeren op variabelen die de uitkomstmaten (mogelijk) beïnvloeden. We stratificeren voor de locatie waaruit geworven wordt en de diagnose (psychotische stoornis of bipolaire stoornis).

Hypotheses worden getoetst d.m.v. regressie analyses, t-toetsen en non-parametrische toetsen. Analyses zijn gebaseerd op het 'intention to treat' principe: iedereen die gerandomiseerd is blijft in de groep waarin hij/zij na randomisatie terecht is gekomen.

Verschillen tussen de experimentele condities onderling en de controlegroep voor wat betreft de secundaire uitkomstmaten zullen explorerend worden geanalyseerd door middel van onder andere multiple regressie analyses. Hierbij zal onder andere onderzocht worden in hoeverre de groepen 1 en 2 verschillen in de mate van vraaggerichtheid en in hoeverre dit samenhangt met het effect van de crisiskaart.

# 10 Haalbaarheid van de studie

Om de haalbaarheid van deze studie te onderzoeken hebben we een peiling verricht onder een groep behandelaars voor patiënten met psychotische stoornissen.

Tijdens een teamvergadering hebben we gevraagd of men bereid is om aan deze studie mee te doen. Ook hebben we gevraagd hoe groot men de kans inschatte dat een patiënt aan de studie mee zou willen doen. Het bleek dat behandelaars (psychiaters, artsen, SPV-ers en andere behandelaars) enthousiast zijn over de studie omdat men het belang van een crisiskaart groot vindt en men is bereid om mee te doen aan de studie.

In hoeverre patiënten ook bereid zijn om mee te doen vindt men moeilijker in te schatten. Eerder onderzoek liet zien dat ongeveer 50% van de patiënten bereid is om aan een dergelijk onderzoek mee te doen (Henderson et al 2004). Volgens de behandelaars lijkt dit reëel. Deelname blijkt vaak erg afhankelijk van de wijze waarop behandelaars en onderzoekers de patiënt benaderen.

We schatten in dat door het enthousiasme van de behandelaars voor deze studie de kans groot is dat we voldoende patiënten kunnen includeren. Indien blijkt dat de werving tegenvalt, bestaat de mogelijkheid om ook andere GGz Instellingen te betrekken bij het onderzoek (b.v. Delta Psychiatrisch Centrum)

## 10.1 Tijdspad

0 - 8 maanden Voorbereidingsfase, informeren behandelaars, selectie patiënten; pilot studie.

9 - 19 maanden Werving patiënten en opstellen crisiskaart.

19 - 37 maanden Follow-up.

37 - 48 maanden Verwerking van de resultaten, analyse en rapportage van de resultaten in een rapport voor ZonMw, artikelen en een proefschrift.

# 11 Ethische overwegingen

## 11.1 Werving en informed consent

De behandelaar informeert de patiënt over het onderzoek. Na toestemming van de patiënt geeft de onderzoeker verdere uitleg over het onderzoek. De onderzoeker benadrukt de vrijwilligheid van deelname en het feit dat weigering van deelname geen enkele verandering zal hebben voor de behandeling van de patiënt.

## 11.2 Randomisatie en weigering

De effecten van het hebben van een crisiskaart zijn nog niet bekend. Daarom lijkt het ons ethisch verantwoord om patiënten willekeurig aan een van de drie condities (crisiskaart opgemaakt met de consulent, of zonder, of geen crisiskaart) toe te wijzen.

Patiënten die deelname aan de randomisatie weigeren, maar toch graag een crisiskaart willen (opgesteld met of zonder crisiskaartconsulent) kunnen dit kenbaar maken. Dit zal dan gebeuren. Zij nemen niet deel aan het onderzoek. Desgewenst kunnen patiënten na het plaatsvinden van de randomisatie alsnog gedurende ieder moment tijdens de studie een crisiskaart opstellen.

# Referenties

Basisberaad Rijnmond. Voorbij de crisis. De ontwikkeling van de crisiskaart in Rijnmond. Uitgave: Basisberaad Rijnmond, Rotterdam 2004

Beller EM, Gebski V, Keech AC. Randomisation in clinical trials. MJA 2002; 177:565-567

Drayton M, Birchwood M, Trower P. Early attachment experience and recovery from psychosis. Brit J Clin Psychology 1998;37:269-284

Gaag M van der. Schizofrenie en posttraumatische stress-stoornis. Dth 2002;22:15-27

Henderson C, Flood C, Leese M, Thornicroft G, Sutherby K, Szmukler G. Effect of joint crisis plans on use of compulsory treatment in psychiatry: single blind randomized controlled trail. BMJ

2004;329:136-138

Mulder CL, Staring ABP, Loos J, Buwalda V, Kuijpers D, Sytema S, Wierdsma AI. De Health of the Nations Outcome Scales in Nederlandse vertaling. Psychometrische kenmerken. Tijdschr Psychiatrie 2004;46:273-285

Mulder CL, Koopmans GT, Lyons JL. Determinants of indicated versus actual level of care in psychiatric emergency services. Psychiatric Serv 2005;56:452-457

Mulder CL, Kroon H (Eds.). Assertive Community Treatment. Wetenschappelijke Uitgeverij Cure & Care Publishers, ISBN 90 76754 44 6, Wageningen, 2005

Mulder CL, Wierdsma AI. Psychiatric disorder, dangerousness and acute involuntary admission in the Netherlands. Opgestuurd voor publicatie

Mulder CL, Wierdsma AI, Broer J, Uitenbroek D. Versnelde stijging van het aantal IBS-en na introductie van de wet BOPZ. Ned Tijdschr Geneeskunde 2006, 150, 6: 319-322.

Mulder CL, Hemert AE. Epidemiologie van de acute psychiatrie. In druk in: Handboek Spoedeisende Psychiatrie, Uitgeverij Benecke N.I. (B)

Mulder CL, Koopmans GT, Selten JP. Dangerousness and Ethnicity as Risk Factors in Emergency Psychiatry. Brit J Psychiatry, 2006, 188, 386-391.

Papageorgiou A, King M, Janmohamed A, Davidson O, Dawson J. Advance directives for patients compulsorily admitted to hospital with serious mental illness. Brit J Psychiatry 2002;181:513-519

Poodt HD. Wiersma AI. Lokale zorgnetwerken: een kwestie van bemoeizorg? Tijdschr

Gezondheidswetenschappen 2001; 79:275-281

Srebnik DS, Rutherford LT, Peto T, Russo, J, Zick E, Jaffe C, Holtzheimer P. The content and clinical utility of psychiatric advance directives. Psychiatric Serv 2005;56:593-598

Sutherby K, Szmukler GI, Halpern A, Alexander M, Thornicroft G, Johnson C, Wright S. A study of crisis cards in a community psychiatric service. Acta Psychiatrica Scand 1999;100:56-61

Swartz MS, Swanson JW, Hannon MJ. Does fear of coercion keep people away from mental health treatment? Evidence from a survey of persons with schizophrenia and mental health professionals. Behaviour Sc Law 2003;21:459-472

Tait L, Birchwood M, Trower P. A new scale (SES) to measure engagement with community mental health services. J Mental Health 2002;11:191-198

Tait L, Birchwood M, Trower P. Predicting engagement with services for psychosis: insight, symptoms and recovery style. Brit J Psychiatry 2003;182:123-128

Thomas P. How should advance statements be implemented. Brit J Psychiatry 2003;182:548-549

Wing JK, Beevor AS, Curtis RH. Health of the Nation Outcome Scale (HONOS). Research and

development. Brit J Psychiatry 1998;172:11-18

| **Naam** | **Handtekening** | **Datum** |
| --- | --- | --- |
| M. Verschuure, directeur divisie Regionale Ketenzorg  namens de verrichter |  |  |
| Prof. dr. C.L. Mulder, projectleider |  |  |

# Bijlage 1 Stroomschema onderzoeksprotocol

# Bijlage 2 Stroomschema randomisatie

# Bijlage 3 Overzicht instrumentarium

#

# Bijlage 4 Onderzoekscontact met patiënt per meetmoment

| **Variabele** | **Vorm** | **Tijd** | **M1** | **M2** | **M3** | **M4** |
| --- | --- | --- | --- | --- | --- | --- |
| Verwachtingen van de crisiskaart | Korte vragenlijst | 5 min | x |  |  |  |
| Veiligheid,controle  over (dreigende) crisis | Korte vragenlijst | 5 min | x | x | x | x |
| Psychisch en sociaal  functioneren | HONoS | 10 min | x | x | x | x |
| Sociale steun | ASR | 5 min | x | x | x | x |
| Ziekteinzicht | Birchwood | 5 min | x | x | x | x |
| Werkrelatie | WAI | 10 min | x | x | x | x |
| Vraaggerichtheid:  -eigen visie, vrij in wat wel/niet gewenst | Te ontwikkelen  Vragenlijst (kort) | 5 min |  | x |  |  |
| Vraaggerichtheid:  - inspraak in beh. | GGZ-Thermometer | 5 min | x | x | x | x |
| Gebruik crisiskaart  (eventueel) | Gestructureerde vragenlijst, te ontwikkelen | 5 min | x | x | x | x |
| Veiligheid,controle  over (dreigende) crisis enz. | Ontwikkelde vragenlijst, kort | 5 min | x | x | x | x |
| Acceptatie van de  ziekte | RSQ | 5 min | x | x | x | x |
|  |  | **Totaal min:** | **60** | **65** | **60** | **60** |

# Bijlage 5 Theoretisch model
